# Prospective observational data informs understanding and future management of Lynch syndrome: insights from the Prospective Lynch Syndrome Database (PLSD)

**DOI:** 10.1007/s10689-020-00193-2

**Published:** 2020-06-08

**Authors:** Toni T. Seppälä, Mev Dominguez-Valentin, Julian R. Sampson, Pål Møller

**Affiliations:** 1 Department of Surgery, Helsinki University Hospital, Helsinki, Finland; 2 Department of Surgical Oncology, Johns Hopkins University, Baltimore, USA; 3 Department of Tumor Biology, Institute of Cancer Research, The Norwegian Radium Hospital, Oslo University Hospital, Oslo, Norway; 4 Division of Cancer and Genetics, Institute of Medical Genetics, Cardiff University School of Medicine, Cardiff, UK

**Keywords:** Lynch syndrome, Hereditary non-polyposis colorectal cancer, Colorectal cancer, Colonoscopy, Surveillance, Prevention, Precision medicine, PLSD, Cancer incidence

## Abstract

The Prospective Lynch Syndrome Database (PLSD) has been developed as an international, multicentre, prospective, observational study that aims to provide age and organ-specific cancer risks according to gene and gender, estimates of survival after cancer and information on the effects of interventions. Recent reports from PLSD provided improved estimates of cancer risks and survival and showed that different time intervals between surveillance colonoscopies did not affect the incidence, stage or prognosis of colorectal cancer. The PLSD reports suggest that current management guidelines for Lynch syndrome should be revised in light of the different gene and gender-specific cancer risks and the good prognosis for the most commonly associated cancers.

In this review, we describe the discrepancies between the current management guidelines for Lynch Syndrome and the most recent prospective observational studies, indicating the areas of further research.

## Lack of big data in Lynch Syndrome: PLSD development

Our understanding of cancer risks and the effects of surveillance in Lynch syndrome (LS) has been informed previously by retrospective studies and limited by the use of clinical criteria to select individuals for molecular testing (Amsterdam I or II criteria, Bethesda guidelines). In addition, the lack of validation in independent cohorts and inconsistent classification of genetic mismatch repair (MMR) variants have mitigated against the calculation of accurate risks which are essential for planning appropriate approaches to the prevention or early diagnosis of cancers.

Researchers from several collaborating European centres agreed to establish the Prospective Lynch Syndrome Database (PLSD) during a meeting in Palma, Mallorca on May 4th 2012. At the time the group was recognized as ‘The Mallorca Group’, but this has more recently expanded and formalized as the European Hereditary Tumour Group (EHTG); a charitable company registered in Scotland (number SC048407). The aim of PLSD is to provide age and organ-specific cancer risks according to gene and gender, describe survival after cancer and determine the effects of interventions. The PLSD aimed at migrating knowledge on LS from expert opinions based on retrospective studies to prospectively obtained empirical observations on cancer incidences, mortality and the effects of interventions in carriers of pathogenic MMR variants (*path_MMR*). A detailed discussion of methods was previously published [1]. Møller’s recent review paper paper [1] summarizes some findings from PLSD that are partially in conflict with what was expected based on previous research, and discusses the emerging knowledge of inherited colorectal cancers including LS during recent decades. Of note, he also examines how to avoid lead-time bias, which was one of the detailed aims of PLSD.

The first PLSD report was published in 2015 and included 1942 MMR carriers followed for 13,782 prospective observation years [2]. In our last report [3], we collected prospective data from a new large cohort of carriers of class 4 and 5 *path_MMR* variants to validate the previous findings. We also updated information on the original cohort to ensure consistent classification of pathogenicity of MMR gene variants. We reported 6350 carriers with 51,646 follow-up years from 18 different countries world-wide that allowed us to derive more precise risk estimates for cancers in LS, categorized by gene and gender [3].

The PLSD findings suggested the need for revision of international clinical guidelines for carriers of *path_MMR* variants in light of the different gene and gender-specific risks and the good prognosis for the most common LS associated cancers.

The cancer risk algorithm at the PLSD website (www.plsd.eu) enables interactive calculation of remaining lifetime risks for cancer in any LS patient by giving their age, gender, and gene variant, thereby facilitating personalized medicine for *path_MMR* carriers.

### Colonoscopy surveillance failed to prevent colorectal cancer

During the last 5 years, prospective observational studies by PLSD [2] and a three-country study by Dutch, Finnish and German researchers [4] have challenged our previous views on LS cancer risks and how to manage them. In these studies, colorectal surveillance by colonoscopy, performed at major clinical academic institutions worldwide, has failed to prevent colorectal cancer (CRC) in carriers of pathogenic variants in the mismatch repair genes *MLH1, MSH2* and *MSH6*. In PLSD, we calculated the lifetime risk of CRC in carriers from 25 to 75 years of age who were receiving colonoscopic surveillance as 43–46% for *path_MLH1* and *path_MSH2* [5]. This risk was validated and refined as being 48–57% (95% confidence interval 41–68%) for *path_MLH1* and 47–51% [39–65%] for *path_MSH2* in a cohort of 6350 *path_MMR* carriers with 51,646 follow-up years [3]. This is in keeping with previously reported prospectively observed incidences of 8.4% [7.1–10.2%] for first CRC and 14.1% [11.5–16.8%] for metachronous CRC after 10 years of follow-up under colonoscopy surveillance [4]. These results have been considered by some to conflict with a previous Finnish follow-up study [6]. That study, however, reported on 10-year incidence and survival, irrespective of the age at which cancer occurred. There may be a later onset of CRC in patients who are followed up by colonoscopy, without a substantial effect on life-time incidence. So far, however, there has been no control group with which to compare the PLSD data in order to test for a right-shift of the cumulative incidence curve without a substantial reduction in the life-time cumulative risk. The PLSD cumulative risks reported do not, however, differ substantially from those derived from retrospective data that were handled statistically by modified segregation analysis. The latter could be considered as providing a risk estimate for past generations who did not undergo regular colonoscopy surveillance. In a retrospective study, Dowty et al. reported risks of 34% [25–50%] and 47% [36–60%] for male *path_MLH1* carriers and *path_MSH2* carriers, and 36% [25–51%] and 37% [27–50%] for female carriers, respectively [7]. As the point estimates from PLSD for prospectively observed CRC risk, starting from initial colonoscopy, excluding prevalent cancers and applying mostly 1–2 year intervals between examinations, were higher than the retrospective risks, these observations tell us that the impact of colonoscopy with polypectomy in preventing CRC is less than was previously thought, based on a controlled study [6, 8].

The difference between the expected and observed results of intervention by colonoscopy has raised numerous questions. In response to the continued occurrence of CRC in patients with LS under surveillance, previous guidelines have recommended shortening the interval between colonoscopies despite the lack of any level 1 evidence to support a benefit to patients, and despite the potential implications for cost-effectiveness and procedure-related adverse events. In fact, the only controlled study reported lower disease stage at detection and better survival in patients receiving 3-yearly colonoscopies than who only had colonoscopies when presenting with symptoms [6]. In the data filed in PLSD and three-country study, shorter 1–2-yearly colonoscopy intervals were associated with neither decreased incidence [4, 9], nor earlier disease stage at detection of CRC [4, 10] and there was no improvement in survival compared to patients having colonoscopy at 3-yearly intervals [11]. These findings raise the intriguing possibility that some CRCs in *path_MMR* carriers may spontaneously disappear: the host immune response may not only remove CRC precursor lesions in *path_MMR* carriers, but may perhaps also remove some infiltrating cancers.

Endoscopists have understandably focused attention on inadequately reported quality indicators of colonoscopy surveillance as a confounding factor in reported studies [12]. This criticism would possibly have greater legitimacy if only a few clinical centers could be shown to be causing the effect through poor quality colonoscopies. While the key performance indicators vary a great deal between randomized controlled endoscopy trials and population-based registry data [4, 13–16], so far there have been no reported inter-observer differences, geographically or by center, in cancer incidence within or between large longitudinal datasets [4]. The reported quality metrics do not support the claim that poor quality colonoscopy, in terms of completeness of the examination or bowel preparation explain the incidence of CRC in LS during surveillance [17]. Differences in adenoma detection rate (ADR) may be explained largely by differences in geography, environment, diet and previous surgery, as well as age, gene, gender and ascertainment variation between the studies. A single ADR threshold cannot be determined to suit all populations. Besides, there are no studies to date showing that a high ADR would prevent CRC and the assumptions underlying ADR as a reliable surrogate marker for cancers prevented may not be true in LS.

Characterization of the molecular pathology of LS-associated CRC has established variability in its pathogenesis that fits fairly well with the findings in clinical observational data. *Path_MLH1* presents with substantially lower adenoma incidence than *path_MSH2*, even though their cumulative CRC incidence is the same [18]. *Path_MLH1*–associated tumors harbor more frequent somatic mutations of *CTNNB1* encoding beta-catenin than *path_MSH2* tumors, suggesting that the non-polypous growth pathway originating from MMR-deficient crypt foci might be more frequent in *path_MLH1* tumors [18]. Similarly, *APC*-mutation associated with progression down the traditional adenoma-carcinoma sequence is more frequent in *path_MSH2*–associated CRCs than in *path_MLH1* [18]. In *path_MSH6*, CRC is less frequent during surveillance [3].

The responses from parts of the health care community to the continuing occurrence of CRC in patients with LS under colonoscopy surveillance has been to increase the frequency of colonoscopy, but without evidence that this will help. At the time, this action appeared to be an educated guess that seemed intuitively correct. However, providers may be conflicted where remuneration is determined by activity (numbers of colonoscopies undertaken) rather than outcomes (cancers prevented or lives extended). It is of note that “choosing wisely” recommendations made by specialist societies usually do not suggest changes in practice that would reduce healthcare use in their own field [19]. Therefore, all societies issuing guidance on colonoscopy interval should critically review their positions. The responsibility of specialist societies is to show the evidence for benefit behind interventions, not to recommend procedures as a precaution simply because there is insufficient evidence. Even when there is evidence of benefit, the costs, opportunity costs and adverse effects of interventions should be considered when formulating clinical guidelines. A number of issues therefore need to be addressed by the LS research community. One immediate priority is the careful review of prospective data to reveal all possible source of bias, as highlighted by Moller [1]. Another is a time trend in the evolution of colonoscopy technology [17]. Health economists must join the field and contribute to the design of upcoming studies in order to produce meaningful information on the cost-effectiveness of different surveillance strategies. If acquiring this requires trials of different intervals between colonoscopies, it will be the duty of specialist societies to facilitate the design and conduct of such studies for the patients their members serve. Such studies will inevitably be large, international, multi-center trials requiring major funding to support hundreds if not thousands of recruits over a long period of follow-up. The ideal would likely be a randomized trial. In the meantime, we will update PLSD data provided by the current contributors: their use of differing national guidelines may, within a few years, generate observational results comparing outcomes between countries that have applied different time intervals between colonoscopies.

### Research and intervention in the management of extra-colorectal cancers may provide an opportunity to gain more quality-adjusted life years

Surveillance for CRC has been very successful in terms of identifying cancers that are subsequently cured. The 10-year crude survival after CRC in the PLSD was more than 90% and was correlated with stage at detection, but not the interval between colonoscopies [3, 11]. The survival pattern has no doubt changed over generations. *path_MMR* carriers now rarely succumb to CRC or endometrial cancer (EC), resulting in an increased incidence and risk of death from later cancers that we are currently unable to downstage or prevent, such as urinary and biliary tract, gastric, pancreatic and ovarian cancers, brain tumors and sarcomas [3, 5, 20]. This risk for new primaries in other organs cannot be described in retrospective studies of former generations where most carriers died from their first cancer.

Despite women with LS having a lifetime risk up to 50% of developing endometrial cancer, there is limited evidence to support gynecological cancer surveillance in females with LS based on the low quality and limited data on surveillance, survival and mortality in the literature. The Manchester consensus conference strongly recommended that risk-reducing total hysterectomy and bilateral salpingo-oophorectomy (BSO) is offered no earlier than 35–40 years of age following completion of childbearing in *path_MLH1*, *path_MSH2* and *path_MSH6* carriers, while there is insufficient evidence to strongly recommend risk-reducing surgery (RRS) for *path_PMS2* carriers [21]. Interrogating the PLSD data, we described the evolving knowledge regarding RRS practice based on gene and age, and we also demonstrated the need for new guidelines and their implementation worldwide in the future that should be based on empirical information on compliance with guidelines and the effects of interventions (Dominguez-Valentin et al. in preparation).

Although the risk of urothelial, prostate and possibly even renal cell cancer is increased, particularly in *path_MSH2* carriers [5], there is hardly any evidence that screening for urothelial cancer would reduce incidence or mortality. One out of four *path_MSH2* carriers develop a urothelial cancer, and one third of those affected die of the cancer [3]. However, we should not repeat the same intuitive fallacies that led to the overuse of colonoscopy. Instead, those working in the field of hereditary cancer should warmly welcome urologists to design and perform studies to create an assessable evidence base for clinical decision-making and guidance regarding early identification and management of urological cancer, especially upper urothelial tract carcinoma. Urologists have insight into over-diagnosis that occurred through wide application of PSA-screening and they are well aware that all interventions need to be carefully tested before being widely incorporated into clinical practice. Screening using genotype-specific targeted imaging might prove beneficial if the sensitivity and specificity could be adjusted to fulfill the criteria of cost-effective precision medicine, for example based on gene, gender, smoking, BMI and family history. The benefit of such intervention would need to be balanced against the different classes of costs incurred, as discussed above for colonoscopic surveillance.

The incidence of gastric cancer has decreased dramatically in the Western countries, and it represents only 2% of all LS-associated cancers worldwide [3]. Routine surveillance for gastric cancer in the LS setting is not considered beneficial, based on several studies [22], and the same applies to small bowel cancer [23, 24]. Future studies should include specific information on cancer histology, numbers of individuals under surveillance that is offered in a research setting, the findings of all examinations and the intervals between examinations [25].

Pancreatic cancer in LS has a poor prognosis compared to the more frequent colorectal and endometrial cancers and is emerging as a significant cause of death in path_MMR carriers who survive their first cancers [1]. We are aware of initiatives that aim to reduce pancreatic cancer incidence or mortality, and more detailed knowledge of pancreatic cancer in LS is a goal for upcoming PLSD studies. Surveillance by endoscopic ultrasound and magnetic resonance cholangiopancreatography has been studied in a longitudinal observational setting and is reported to confer some survival benefit for hereditary cancers found by screening. However, none of the early lesions in the program were diagnosed in LS patients [26]. There is currently no evidence to support invasive or non-invasive surveillance for pancreatic cancer in clinical practice in LS.

Although, in LS, no surveillance strategies for cancers other than colorectal cancers are of proven value, some of them are recommended by health care professionals. As the number of living *path_MMR* carriers increases due to better survival and an increase in genetic testing, a multidisciplinary approach is required to design studies that will inform decisions about which screening procedures these patients should be offered. Their healthcare should be based on empirical information on effects of interventions and their cost-effectiveness.

## Current research using PLSD

We will update the data held by PLSD to describe more precisely CRC risk, stage and survival stratified by the interval between colonoscopies. We will describe the uptake of risk-reducing gynecological surgery by age and gene, and we are in the process of describing risk estimates for cancer in LS categorized by *path_MMR* variant class.

Although no significant geographical differences were identified between the original PLSD cohort recruited in Europe but excluding Germany [5] and the validation cohort recruited mainly in Germany, the Americas, and Australasia [3], we are aware that differences in follow-up practices might impact cancer risks. There is a need to study the geographical differences in CRC and cancers in the other organs.
